# A novel LC-MS/MS method for simultaneous estimation of acalabrutinib and its active metabolite acalabrutinib M_27_ in human plasma and application to a human pharmacokinetic study†

**DOI:** 10.1039/d1ra09026g

**Published:** 2022-02-25

**Authors:** Venkat Rao Valluri, Naresh Kumar Katari, Chirag Khatri, Pankaj Kasar, Srinivasa Rao Polagani, Sreekanth Babu Jonnalagadda

**Affiliations:** Department of Chemistry, School of Science, GITAM Deemed to be University Hyderabad 502329 India nkatari@gitam.edu +91-9177712000; AnaCipher Clinical Research Organization Hyderabad-500013 India; School of Chemistry & Physics, College of Agriculture, Engineering & Science, Westville Campus, University of KwaZulu-Natal P Bag X 54001 Durban-4000 South Africa Jonnalagaddas@ukzn.ac.za +27837854344

## Abstract

A simple, specific, selective and accurate bioanalytical method was developed and validated for simultaneous estimation of acalabrutinib and its active metabolite in human plasma using liquid chromatography-tandem mass spectrometry (LC-MS/MS). Deuterated analogs of both the analytes were used as internal standards. The extraction of analytes and internal standards were evaluated from the human plasma by liquid–liquid extraction technique using methyl tertiary butyl ether (TBME). The separation of the analytes was carried out on Zorbax Eclipse XDB-C_18 (_150 × 4.6 mm, 5 μm) column with a mixture of acetonitrile and 10 mM ammonium formate in 0.1% formic acid buffer (65 : 35, v/v) as mobile phase at a flow rate of 1 mL min^−1^. The method linearity was determined in the widen concentration range from 5.000 ng mL^−1^ to 1600 ng mL^−1^ with *r*^2^ > 0.99. The entire method validation was carried out as per the USFDA guidelines on bioanalytical method validation and all validation experiment results were found within acceptable limits. Clinical pharmacokinetic study of both the parent drug and its active metabolite was successfully performed on six healthy volunteers under fasting conditions by applying the present method.

## Abstract 2

A simple, specific, selective and accurate bioanalytical method was developed and validated for simultaneous estimation of acalabrutinib and its active metabolite in human plasma using liquid chromatography-tandem mass spectrometry (LC-MS/MS).

## Graphical abstract

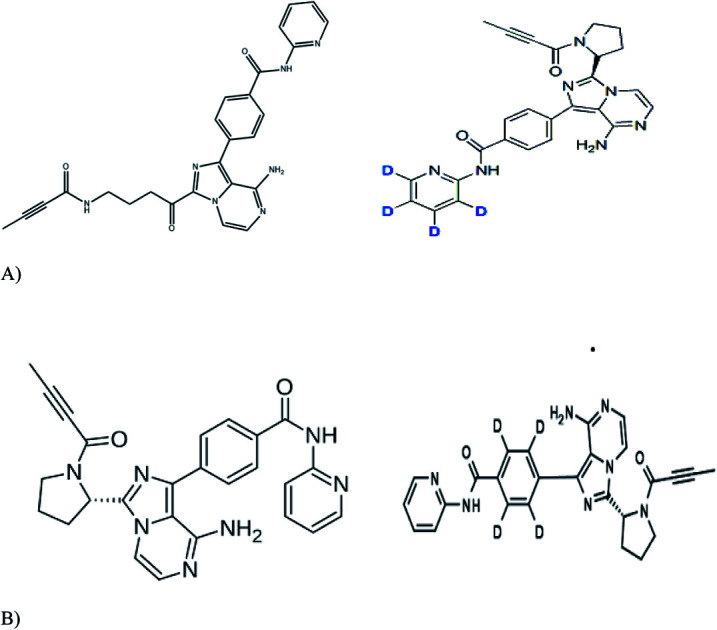

## Introduction

Bruton's tyrosine kinase (BTK) is a promising drug target for malignancies of B cells. BTK is a signaling molecule of the B cell antigen receptor (BCR) and cytokine receptor pathways.^1,2^ Acalabrutinib (chemically (*S*)-4-(8-amino-3-(1-but-2-ynoylpyrrolidin-2-yl)-imidazo[1,5-α] pyrazin-1-yl)-*N*-(pyridin-2-yl)-benzamide) is a covalent inhibitor of BTK, used to treat chronic lymphocytic leukemia (CLL) in adults.^3–5^ A metabolite of acalabrutinib (acalabrutinib M_27_; chemically 4-[8-amino-3-[1-oxo-4-[(1-oxo-2-butyn-1-yl) amino] butyl] imidazole [1, 5-α] pyrazin-1-yl]-*N*-2-pyridinyl-benzamide) produced by CYP3A4 mediated oxidation is also pharmacologically active and selectively inhibits BTK.^6,7^

Acalabrutinib is a small molecule inhibitor of BTK. Acalabrutinib and its active metabolite, ACP-5862 form a covalent bond with a cysteine residue in the BTK active site, leading to inhibition of BTK enzymatic activity. BTK is the signaling molecule of the B cell antigen receptor (BCR) and cytokine receptor pathways. In B cells, BTK signaling results in activation of pathways necessary for B-cell proliferation, trafficking, chemotaxis, and adhesion. In non-clinical studies, acalabrutinib inhibited BTK mediated activation of downstream signaling proteins CD86 and CD69 inhibited malignant B-cell proliferation and tumor growth in mouse xenograft models.^8^

As per the literature, two bioanalytical methods based on LC-MS/MS for estimation of acalabrutinib have been reported.^9,10^ The LC-MS/MS method reported by Zheli Jiang *et al.*^9^ is to estimate ACB, ibrutinib and their metabolites in dog plasma. The other method reported by Shruti Surendran *et al.*^10^ is to quantify ACB in rat plasma. However, both the methods are applicable only for preclinical studies in animal designs. The reported methods^11–13^ are also not suitable for determination of ACB and ACBM in human plasma for clinical application. To the best of our knowledge, there is no bioanalytical method reported for simultaneous estimation of ACB and its active metabolite ACBM in human plasma. The objective of the current study is to develop simple, specific, selective, accurate and economical LC-MS/MS method for simultaneous quantification of ACB and ACBM in human plasma and to perform pharmacokinetic study of both the analytes in humans. The current method is first LC-MS/MS method for the simultaneous determination of ACB and ACBM in human plasma with clear experimental procedure to support the human pharmacokinetic studies.

## Experimental

### Materials and reagents

**Fig. 1:**
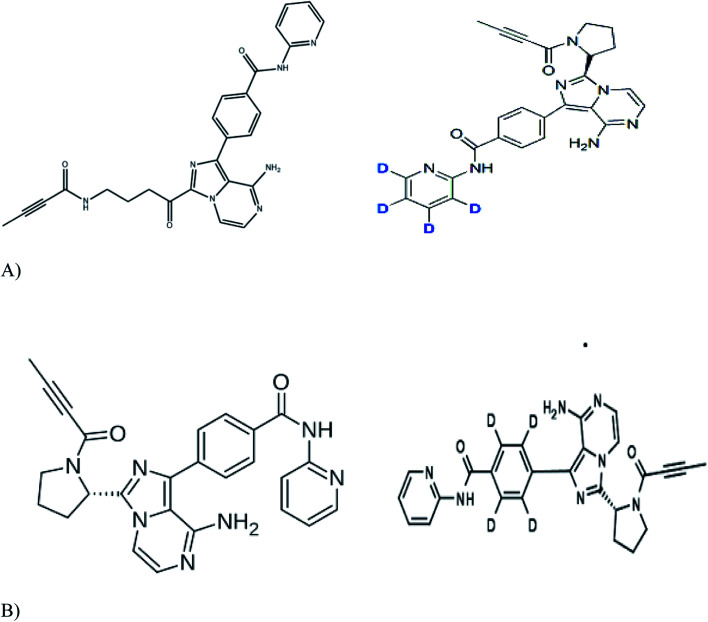
Molecular structures of (A) acalabrutinib (left panel) and acalbrutinib-D4 (right panel) (B) acalabrutinib metabolite M27 (left panel) and acalabrutinib metabolite M_27_-D4 (right panel).

Working standards of acalabrutinib (98.79%), acalabrutinib-D_4_ (100%) and M_27_ metabolite acalabrutinib-D_4_ (98.59%) were obtained from Simson Pharma Limited (Mumbai, India) and acalabrutinib M_27_ (96.82%) was purchased from Diacel Chilar Technologies (Hyderabad, India) (Fig. 1 for molecular structures). HPLC grade water and analytical grade formic acid, dimethyl sulfoxide (DMSO) and TBME were procured from Rankem (Gurugram, India). HPLC grade acetonitrile was procured from RCI Labscan Ltd (Bangkok, Thailand). Ammonium formate of analytical grade was procured from Loba Chemie Pvt Ltd (Mumbai, India). Human blank plasma was obtained from Laxmi Sai Clinicals (Hyderabad, India).

### Instrument and conditions

**Fig. 2:**
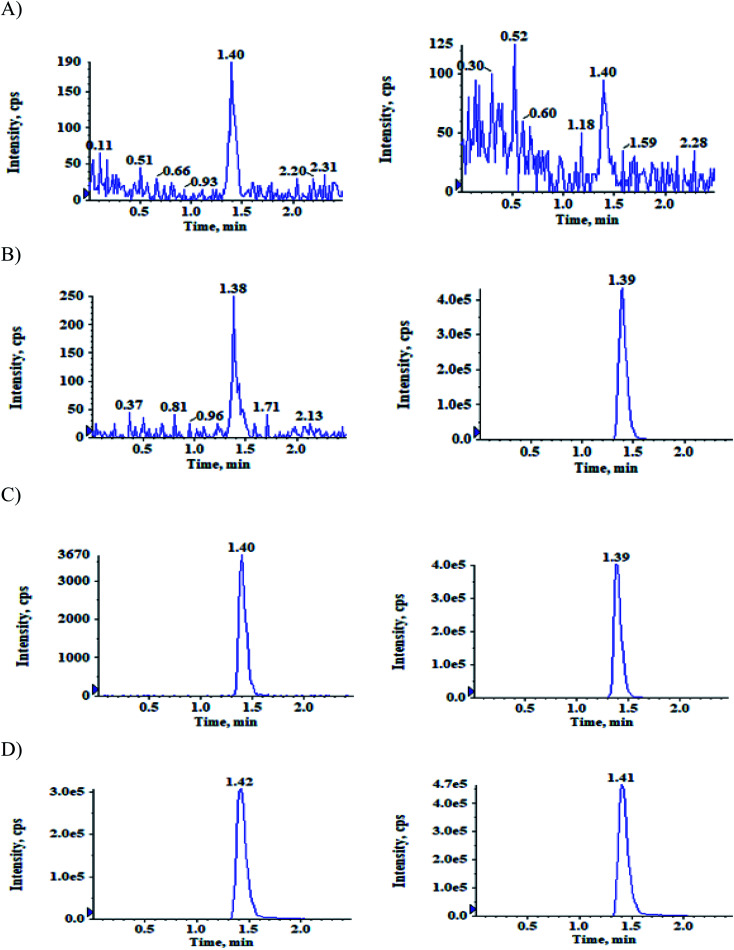
Typical MRM chromatograms of ACB (left panel) and IS-1 (right panel) in (A) double blank plasma (B) blank plasma with IS-1 (C) LLOQ QC sample and (D) subject sample after 0.83 h of administration of 100 mg oral dose of ACB.

The quantitative analysis was performed using Shimadzu LC-20AD coupled with mass spectrometer AB SCIEX API-4500 (Foster city, CA, USA) interfaced *via* Turbo ion spray™. After sample extraction an aliquot of 15 μL was injected into Zorbax Eclipse XDB-C_18_ (150 × 4.6 mm, 5 μm; Agilent technologies, CA, USA) column mobile phase (mixture of acetonitrile and 10 mM ammonium formate in 0.1% formic acid buffer, 65 : 35, v/v) was used to separate analytes from the internal standards and matrix components well in isocratic mode with a flow rate of 1 mL min^−1^. Mass spectrometer in positive ion mode was used for quantification of the separated components. Multiple Reaction Monitoring (MRM) mode was used for monitoring precursor product ion transitions at *m/z* 466.1–372.1 for ACB, 482.1–388.1 for ACBM, 470.1–376.1 for IS-1 and 486.1–388.1 for IS-2 (Fig. 2). Analyst software version 1.7.2 was used for processing the analytical data. Source dependent parameters were optimized for the analytes and internal standards at the following intervals, ion spray voltage: 5500 V, turbo heater temperature: 550 °C, curtain gas: 40 psi, collision activation dissociation: 7 psi, nebulizer gas: 35 psi and auxiliary gas: 40 psi. The compound-dependent parameters maintained were entrance potential: 10 V, declustering potential: 80 V, collision energy: 37 V and collision cell exit potential: 12 V for all the analytes and internal standards. Q1 and Q3 quadrupoles were set at unit resolution.

### Preparation of calibration curve standards and quality control samples

Prepared primary stock solutions (1 mg mL^−1^) each of ACB, ACBM, acalabrutinib-D_4_ (IS-1) and M_27_ metabolite acalabrutinib-D_4_ (IS-2) in DMSO. Two sets were prepared individually for the analytes, one for calibration curve standards (CC) and the other for quality control samples (QC). Working solutions of the analytes were prepared using a mixture of acetonitrile and water (60 : 40, v/v; diluent) from two sets, one for CC and the other for QC. A combined working solution of IS-1 and IS-2 (10 000 ng mL^−1^ each) was prepared in the same diluent.

950 μL of K_2_ EDTA human plasma aliquoted with 50 μL of appropriate working solution to prepare CC standards of both the analytes at a concentration of 5.045, 10.090, 25.224, 60.057, 142.992, 317.760, 635.520, 960.000, 1280.000 and 1600.00 ng mL^−1^. QC samples were also prepared at concentrations of 5.065 ng mL^−1^ (LLOQQC, lower limit of quantitation quality control), 14.227 ng mL^−1^ (LQC, low quality control) 177.840 ng mL^−1^ (MQC-1, medium quality control-1), 555.750 ng mL^−1^ (MQC-2) and 1235.000 ng mL^−1^ (HQC, high quality control) for both the analytes. The spiked plasma samples were stored at −70 ± 10 °C for intended usage.

### Sample preparation

Thawing of the samples was done at room temperature, followed by vortexed. To 200 μL of plasma, 20 μL of the combined internal standard working solution was added in RIA vial, then vortexed again. To the above, 2.5 mL of TBME was added and vortexed for 10 min at 2000 rpm. Centrifugation was done at 4000 rpm for 10 min at 4 °C and the supernatant was collected and evaporated to dryness at 40 °C under a gentle stream of nitrogen. The samples were reconstituted using 1 mL of mobile phase and 15 μL injected into the instrument.

## Method validation

As part of method validation following experiments were carried out as per the USFDA guidelines on bioanalytical method validation.^14^ The selectivity, specificity, sensitivity, matrix effect, linearity, precision, accuracy, recovery, dilution integrity, aqueous and matrix stabilities were evaluated.

### Pharmacokinetic study design

After completion of method validation, the method was applied to study the pharmacokinetics study of ACB and ACBM on six healthy male subjects. The pharmacokinetic study was approved by an institutional ethics committee (Vasavi ethics committee, Hyderabad, Reference No: 003-2021). Informed consent was obtained from all the study volunteers. A single oral dose of ACB capsule 100 mg was administered under fasting conditions to subject. Blood samples of 4 mL were collected each time at 0.0 (pre-dose), 0.08, 0.17, 0.25, 0.33, 0.42, 0.50, 0.67, 0.83, 1.00, 1.25, 1.50, 1.75, 2.00, 2.50, 3.00, 4.00, 6.00, 8.00, 12.00, 16.00 and 24.00 h after administration of the ACB capsules orally into labeled vacutainer collection tubes containing K_2_EDTA as an anticoagulant. All the sample tubes were centrifuged at 4 °C for 10 min at 4000 rpm and the collected plasma samples were stored at −70 ± 10 °C till their usage. The samples were then processed and analysed using the developed method and the PK parameters were calculated using WinNonlin® software version 8.3 (Pharsight Corporation, USA) by non-compartmental model. Incurred sample reanalysis was done to ensure the reproducibility of the method by analyzing few samples from each subject, one near *C*_ma*x*_ and the other samples at elimination phase.

## Results and Discussion

As per the literature, there were no methods reported for the estimation of ACB and ACBM in human plasma. The objective of the study is to develop a selective, sensitive, specific and accurate method to estimate ACB and its active metabolite ACBM simultaneously using LC-MS/MS. Electrospray ionization was used in both positive and negative modes and the intensity of both the analytes and internal standards was high with positive mode and then selected. Obtained the most sensitive transitions at *m/z* 466.1–372.1 for ACB, 482.1–388.1 for ACBM, 470.1–376.1 for IS-1 and 486.1–388.1 for IS-2.

The chromatographic conditions optimized were column, mobile phase, and flow rate to attain satisfactory separation and resolution from endogenous components of plasma and get good peak symmetry and response within a minimal run time. Tried varying strengths of ammonium formate buffer containing acid additives of acetic acid or formic acid with HPLC grade acetonitrile on Kromasil 100-C_18_, 100 × 4.6 mm, 5 μm; Ace Phenyl Column, 150 × 4.6 mm, 5 μm; Zorbax Eclipse XDB-C_18,_ 150 × 4.6 mm, 5 μm and Zorbax XDB-Phenyl, 75 × 4.6 mm, 3.5 μm columns at different flow rates. The retention time of ACB and IS-1 was 1.45 ± 0.3 min and ACBM and IS-2 was 1.60 ± 0.3 min, allowing a short run time of 2.50 min.

The sample extraction was initially carried out using protein precipitation with acetonitrile. However, the recovery was feeble and inconsistent. Later, samples were extracted using solid phase extraction (SPE) on Strata-X™ 33 μm polymeric sorbent cartridges which has provided low recovery of both the analyte and metabolites. Liquid–Liquid extraction of the sample with ethyl acetate and *tert* butyl methyl ether was consistent with good recovery for analyte and metabolites including internal standards with good peak shape even at LLOQ QC level.

Deuterated analogs are preferred in bioanalytical methods as they mimic the analytes in extraction, separation and ionization. Hence, in the present study deuterated analogs of the analytes were used as internal standards.

Tried liquid–liquid extraction to extract the analytes from the plasma using ethyl acetate, TBME and dichloromethane. The recovery with TBME was reproducible and the response was also acceptable. Hence, LLE with TBME was selected for sample extraction.

### Carryover

Evaluated the carryover effect by measuring the peak areas of blank samples following an upper limit of quantification (ULOQ-1600 ng mL^−1^) sample to the analyte area of newly injected LLOQ standard. The carryover at both analyte and internal standard retention times were below 20% and 5% respectively.

### Selectivity

**Fig. 3:**
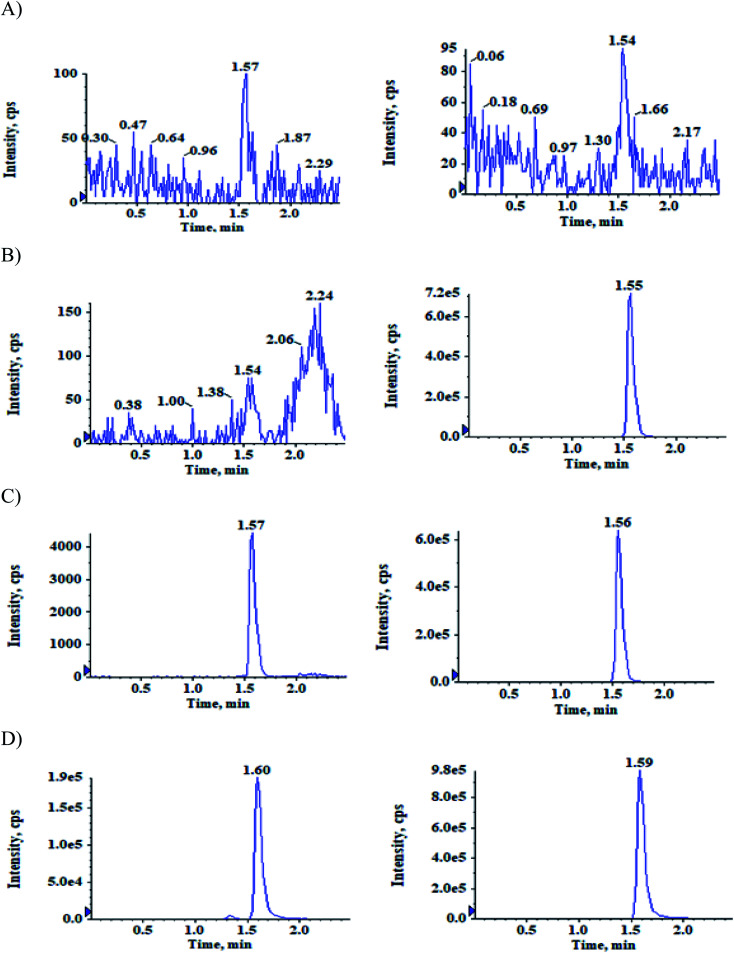
Typical MRM chromatograms of ACBM (left panel) and IS-2 (right panel) in (A) double blank plasma (B) blank plasma with IS-2 (C) LLOQ QC sample and (D) subject sample after 0.83 h of administration of 100 mg oral dose of ACB.

The selectivity relative to the endogenous matrix was evaluated using non pooled blank human plasma and plasma spiked with internal standards to confirm the interference at both analyte and internal standard retention times. There was no interference of endogenous components at the retention times of the analytes and the internal standards (Fig. 2 and 3). Also, internal standards did not interfere directly with the MRM channel of the analytes.

### Specificity

The potential interference at the peak regions of ACB, ACBM and its internal standards were evaluated as part of the specificity experiment to assess the interference at respective retention times contributed from analytes to ISTDs and *vice versa*. No significant cross-interference contributed from analytes to ISTDs and *vice versa*.

### Matrix effect

Eight lots of human plasma along with one lot of lipemic and hemolysed plasma samples at LQC and HQC levels were extracted and injected on LC-MS/MS instrument to study the effect of matrix ions as the IS normalized matrix factors were found to be in the range of 0.97 to 1.06 for both the analytes.

### Linearity and sensitivity

Calibration curves were generated in the concentration range of 5.045 ng mL^−1^ to 1600.000 ng mL^−1^ using least squares regression model of 1/*x*^2^. The correlation coefficient values (*r*^2^) were found to be in the range of 0.9966 to 0.9978.

Found the method to be sensitive with signal to ratio (S/N) value to be ≥10 for the LLOQ QC level of the analytes.

### Precision and accuracy

Intra and inter-day precision and accuracy were evaluated by analyzing six batches of QC samples at five concentration levels *i.e.* LLOQ QC, LQC, MQC-1, MQC-2 and HQC. The accuracy results were found to be 90.92 to 101.12% with precision (%CV) range of 1.43 to 4.46% during intra-day analysis for ACB and 93.89 to 100.76% with precision (%CV) range of 1.43 to 4.42% during intra-day analysis for ACBM. The accuracy results were found to be 92.95 to 101.12% with precision (%CV) range of 1.59 to 6.39% during inter-day analysis for ACB and 94.91 to 100.17% with precision range 1.77 to 4.68% for ACBM (refer Table 1).

Intra-day and inter-day precision and accuracy

**Table:** 

Analyte	QC	Conc. spiked (ng mL^−1^)	Intra-day precision and accuracy (*n* = 12; 6 from each batch)			Inter-day precision and accuracy (*n* = 18; 6 from each batch)		
			Conc. found (mean; ng mL^−1^)	Accuracy (%)	Precision (%CV)	Conc.Found (mean; ng mL^−1^)	Accuracy (%)	Precision (%CV)
ACB	LLOQ QC	5.05	4.78 ± 0.21	94.76	4.46	4.85 ± 0.31	96.02	6.39
	LQC	14.42	13.11 ± 0.34	90.92	2.57	13.40 ± 0.68	92.95	5.10
	MQC-1	180.26	171.70 ± 4.47	95.25	2.60	170.64 ± 4.40	94.66	2.58
	MQC-2	563.31	569.64 ± 13.85	101.12	2.43	569.60 ± 11.40	101.12	2.00
	HQC	1251.80	1216.00 ± 20.21	97.14	1.66	1213.19 ± 19.30	96.92	1.59
ACBM	LLOQ QC	5.06	5.02 ± 0.21	99.29	4.11	4.94 ± 0.23	97.65	4.68
	LQC	14.46	13.58 ± 0.60	93.89	4.42	13.72 ± 0.64	94.91	4.63
	MQC-1	180.74	176.71 ± 3.70	97.77	2.09	174.98 ± 4.41	96.82	2.52
	MQC-2	564.81	569.08 ± 8.97	100.76	1.58	565.77 ± 10.03	100.17	1.77
	HQC	1255.13	1232.90 ± 17.59	98.23	1.43	1220.97 ± 26.99	97.28	2.21

**Table:** 

Matrix effect			
	QC	Precision (%)	IS normalized factor
ACB	LQC	2.26	0.98–1.04
	HQC	1.77	0.98–1.03
ACBM	LQC	2.26	0.98–1.04
	HQC	1.77	0.98–1.03

### Recovery and dilution integrity

The % recovery obtained at LQC, MQC-2 and HQC levels were 92.75%, 82.50% and 83.31% for ACB and 100.43%, 92.52% and 95.54% for ACBM respectively, when calculated using mean area response of extracted samples and aqueous samples. For internal standards, the % recovery obtained was 93.55% and 105.63% for IS-1 and IS-2 respectively.

At four times dilution (5118.706 ng mL^−1^), the precision (%CV) and accuracy were 2.25% and 97.57% for ACB and 1.81% and 98.08% for ACBM, respectively.

#### Stability studies

Stability studies *viz.* BTS (benchtop stability), ASS (auto-sampler stability), FT (freeze-thaw stability), RI (re-injection stability), WES (wet extract stability), STS (short-term stability) and LTS (long-term stability) were carried out at LQC and HQC levels. The results obtained were within the acceptance limits (Table 2).

**Table:** Stability data

Analyte	Stability	Storage condition	Level	Conc. spiked (ng mL^−1^)	Conc. found (mean; ng mL^−1^)	%Stability	Precision (%CV)
ACB	Wet extract stability	Room temperature (49 h)	LQC	14.42	13.83	99.62	1.20
			HQC	1251.80	1227.27	99.40	2.06
	Re-injection stability	69 h at 10 °C	LQC	14.42	13.04	99.05	2.14
			HQC	1251.80	1222.20	99.61	7.03
	Auto-sampler stability	Auto-sampler temperature (10 °C; 55 h)	LQC	14.42	13.83	99.63	3.43
			HQC	1251.80	1219.59	98.78	2.67
	Freeze–thaw stability	After 4th cycle at −70 ± 10 °C	LQC	14.42	13.80	99.41	2.23
			HQC	1251.80	1214.19	98.34	2.89
	Short term stability	3 days at −20±5 °C	LQC	14.42	13.51	97.32	3.30
			HQC	1251.80	1246.11	100.93	2.41
	Long term stability	70 days at −70 ± 10 °C	LQC	14.42	14.08	99.62	1.30
			HQC	1251.80	1236.28	100.56	
	Bench top stability	Room temperature (24 h)	LQC	14.42	13.63	98.25	2.52
			HQC	1251.80	1206.79	97.74	1.92
ACBM	Wet extract stability	Room temperature (49 h)	LQC	14.46	13.76	99.14	2.09
			HQC	1255.13	1214.89	98.55	1.96
	Re-injection stability	69 h at 10 °C	LQC	14.46	13.59	97.94	4.07
			HQC	1255.13	1196.78	96.57	3.03
	Auto-sampler stability	Auto-sampler temperature (10 °C; 55 h)	LQC	14.46	13.59	97.92	1.00
			HQC	1255.13	1214.76	98.54	2.64
	Freeze–thaw stability	After 4th cycle at −70 ± 10 °C	LQC	14.46	13.77	99.20	2.46
			HQC	1255.13	1212.37	98.34	1.46
	Short term stability	3 days at −20±5 °C	LQC	14.46	14.10	101.56	2.64
			HQC	1255.13	1232.18	99.95	0.84
	Long term stability	70 days at −70 ± 10 °C	LQC	14.46	13.61	98.62	1.62
			HQC	1255.13	1224.47	99.66	0.84
	Bench top stability	Room temperature (24 h)	LQC	14.46	13.90	100.15	2.88
			HQC	1255.13	1216.90	98.71	0.97

### Pharmacokinetic study

**Fig. 4:**
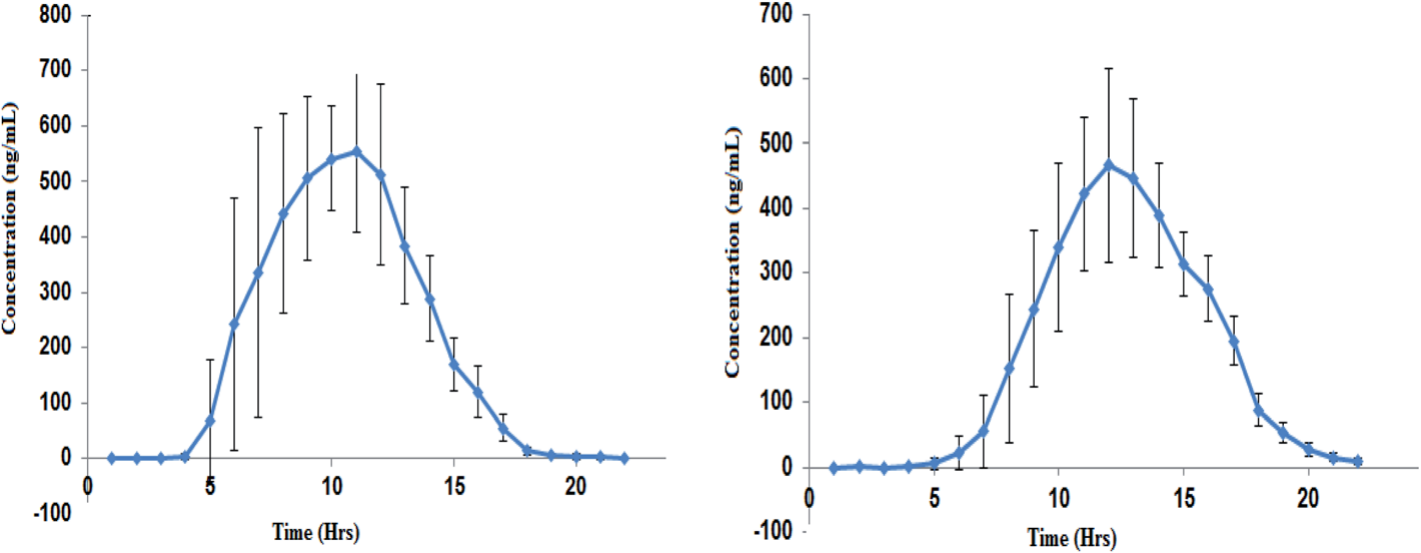
Mean (±SD) plasma concentration–time profile of ACB (left panel) and ACBM (right panel) following oral dose of 100 mg ACB capsule.

The developed method was applied to estimate the concentration of ACB and ACBM present in the plasma of the volunteers after administration of ACB capsule 100 mg. From the data obtained PK parameters were calculated (Table 3) by plotting the mean plasma concentration–time profile (Fig. 4).

**Table:** Pharmacokinetic parameters data

Parameter	ACB (mean ± SD)	ACBM (mean ± SD)
*C*_ma*x*_ (ng mL^−1^)	638.14 ± 81.31	495.03 ± 136.55
*T*_max_ (h)	1.03 ± 0.37	1.50 ± 0.22
AUC_0→*t*_ (ng h mL^−1^)	1141.88 ± 228.00	1850.63 ± 334.49
AUC_0→*∞*_ (ng h mL^−1^)	1156.10 ± 225.99	1914.48 ± 352.99
*T*_1/2_ (h)	2.44 ± 1.69	5.03 ± 0.45
Kel (h^−1^)	0.43 ± 0.26	0.14 ± 0.014

The reanalysis of incurred samples confirmed the method reproducibility for 20 samples near the *C*_max_ and at the elimination phase from all the analyzed subjects. The difference between original and repeat concentrations was below 15%. The results of ISR samples are tabulated in (Table 4).

**Table:** Incurred sample reanalysis data of ACB and ACBM

ACB					ACBM				
Sample no.	Initial conc. (ng mL^−1^)	Re-assay conc. (ng mL^−1^)	Mean	% Difference	Sample No	Initial conc. (ng mL^−1^)	Re-assay conc. (ng mL^−1^)	Mean	% Difference
1	837.751	804.051	820.901	−4.11	1	373.691	347.070	360.381	−7.39
2	46.858	41.768	44.313	−11.49	2	24.379	22.403	23.391	−8.45
3	845.859	794.645	820.252	−6.24	3	356.297	323.025	339.661	−9.80
4	47.133	43.748	45.441	−7.45	4	28.936	26.071	27.504	−10.42
5	854.003	827.950	840.977	−3.10	5	687.124	714.349	700.737	3.89
6	70.779	68.194	69.487	−3.72	6	29.814	28.734	29.274	−3.69
7	726.478	745.598	736.038	2.60	7	653.701	664.139	658.920	1.58
8	46.799	45.321	46.060	−3.21	8	29.093	29.437	29.265	1.18
9	1026.883	1059.336	1043.110	3.11	9	642.853	643.522	643.188	0.10
10	35.759	32.883	34.321	−8.38	10	22.711	22.510	22.611	−0.89
11	1377.927	1384.875	1381.401	0.50	11	724.561	725.367	724.964	0.11
12	32.405	30.940	31.673	−4.63	12	20.610	18.596	19.603	−10.27
13	629.632	640.821	635.227	1.76	13	547.154	563.771	555.463	2.99
14	42.964	40.491	41.728	−5.93	14	16.320	16.881	16.601	3.38
15	770.710	816.710	793.710	5.80	15	662.054	681.726	671.890	2.93
16	37.706	34.705	36.206	−8.29	16	16.992	15.321	16.157	−10.34
17	744.784	745.829	745.307	0.14	17	389.451	372.408	380.930	−4.47
18	52.478	47.543	50.011	−9.87	18	17.075	15.627	16.351	−8.86
19	1058.196	1083.198	1070.697	2.34	19	468.985	451.059	460.022	−3.90
20	20.195	19.934	20.065	−1.30	20	15.593	15.430	15.512	−1.05

## Conclusions

The bioanalytical method for simultaneous of ACB and ACBM in human plasma was developed using LC-MS/MS technique and fully validated as per USFDA bioanalytical method validation guidelines. Reproducible and reliable results were produced by the use of deuterated analogues of the analytes as internal standards. The method is highly appropriate for the analysis of ACB and ACBM simultaneously within a minimal run time of 2.50 min. The current method is superior to the reported methods with respect to novelty, widened calibration curve range, detailed experimental procedure, low run time with minimal sample processing volume and pharmacokinetic study on healthy human subjects that are supplementary advantages for the evaluation of pharmacokinetic parameters. Confirmed the reproducibility of method based on the incurred sample reanalysis results. None of the calibration curve standards and quality controls samples failed to meet the acceptance criteria. The present method's selectivity, specificity and sensitivity proved for its suitability in human pharmacokinetic and bioequivalence studies.

## Author contributions

All the authors are responsible for the following: study conception and design, data collection, analysis, interpretation of results and preparation of the manuscript. All the authors reviewed the results and approved the final manuscript.

## Conflicts of interest

There are no conflicts to declare.

## Supplementary Material

RA-012-D1RA09026G-s001

RA-012-D1RA09026G-s002
