# Supplementary material for: A novel LC-MS/MS method for simultaneous estimation of acalabrutinib and its active metabolite acalabrutinib M27 in human plasma and application to a human pharmacokinetic study
Source: RSC Adv. 2022 Feb 25;12(11):6631–9. doi: 10.1039/d1ra09026g (PMC8982068; doi:10.1039/d1ra09026g)
Supplement: RA-012-D1RA09026G-s002 [file RA-012-D1RA09026G-s002.pdf]

Electronic Supplementary Material (ESI) for RSC Advances.
This journal is © The Royal Society of Chemistry 2022

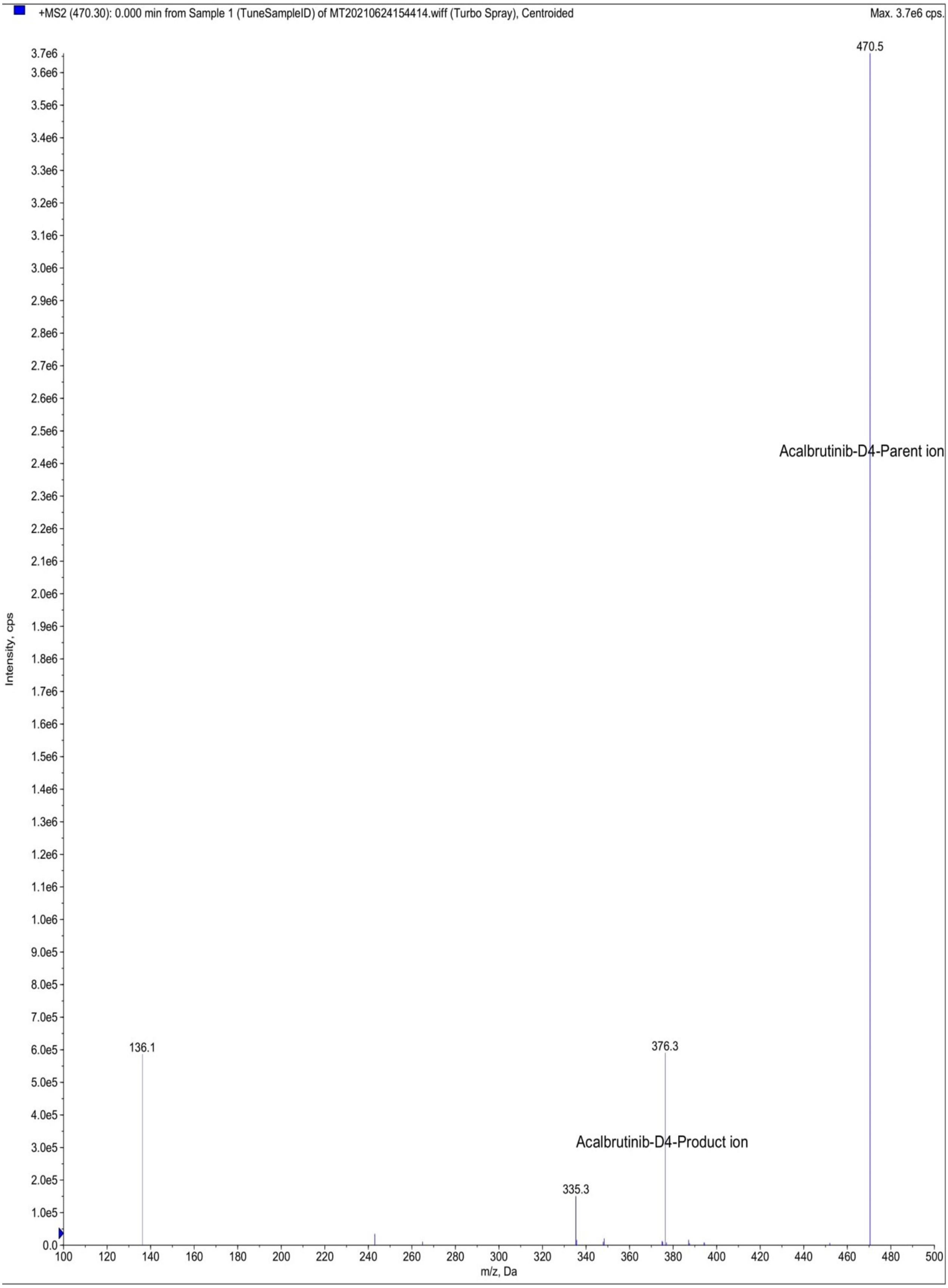

A)

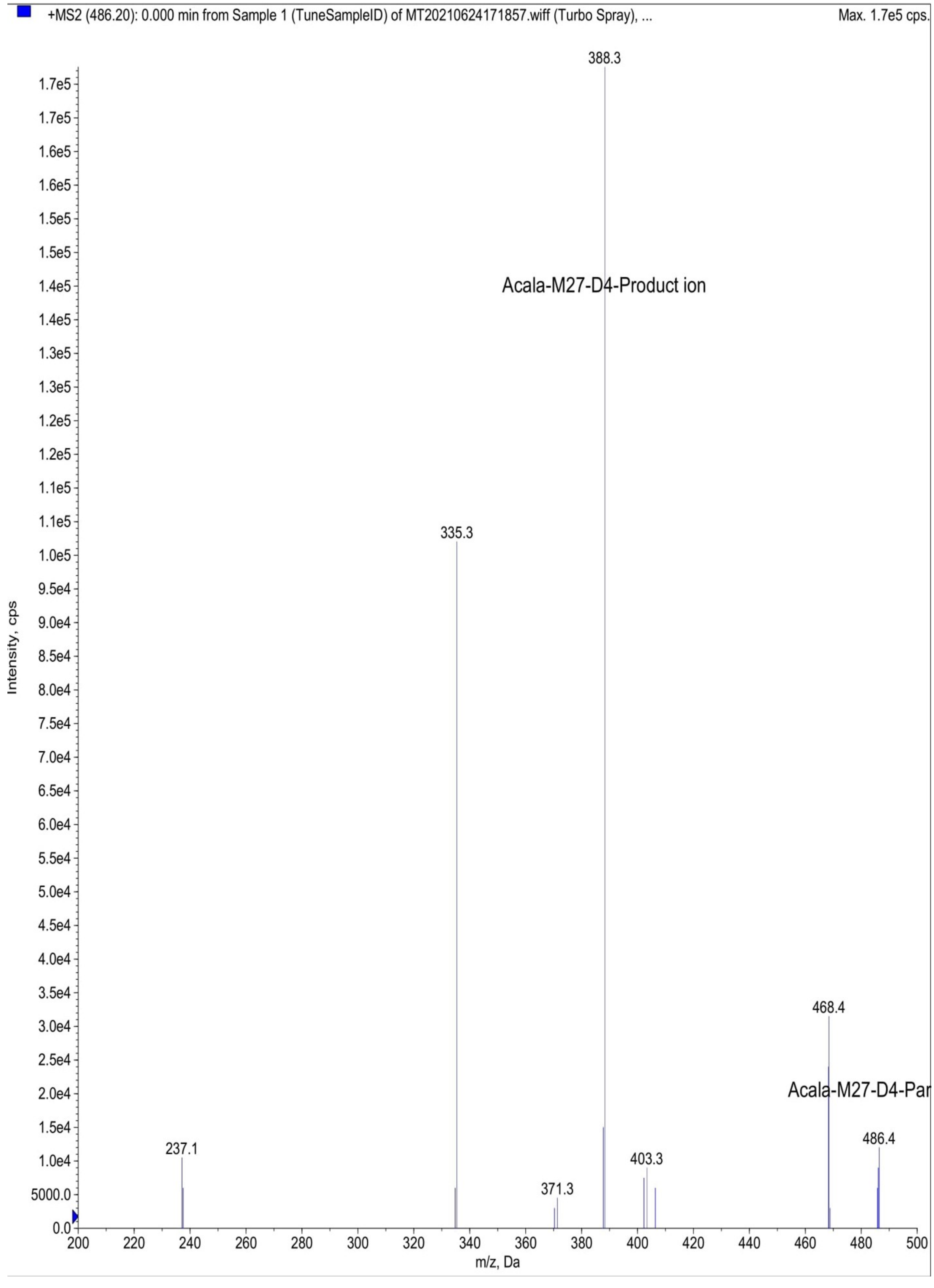
+MS2 (486.20): 0.000 min from Sample 1 (TuneSampleID) of MT20210624171857.wiff (Turbo Spray), ...
Max. 1.7e5 cps.
Acala-M27-D4-Product ion
Acala-M27-D4-Par
388.3
335.3
468.4
486.4
237.1
403.3
371.3
Intensity, cps
m/z, Da

B)

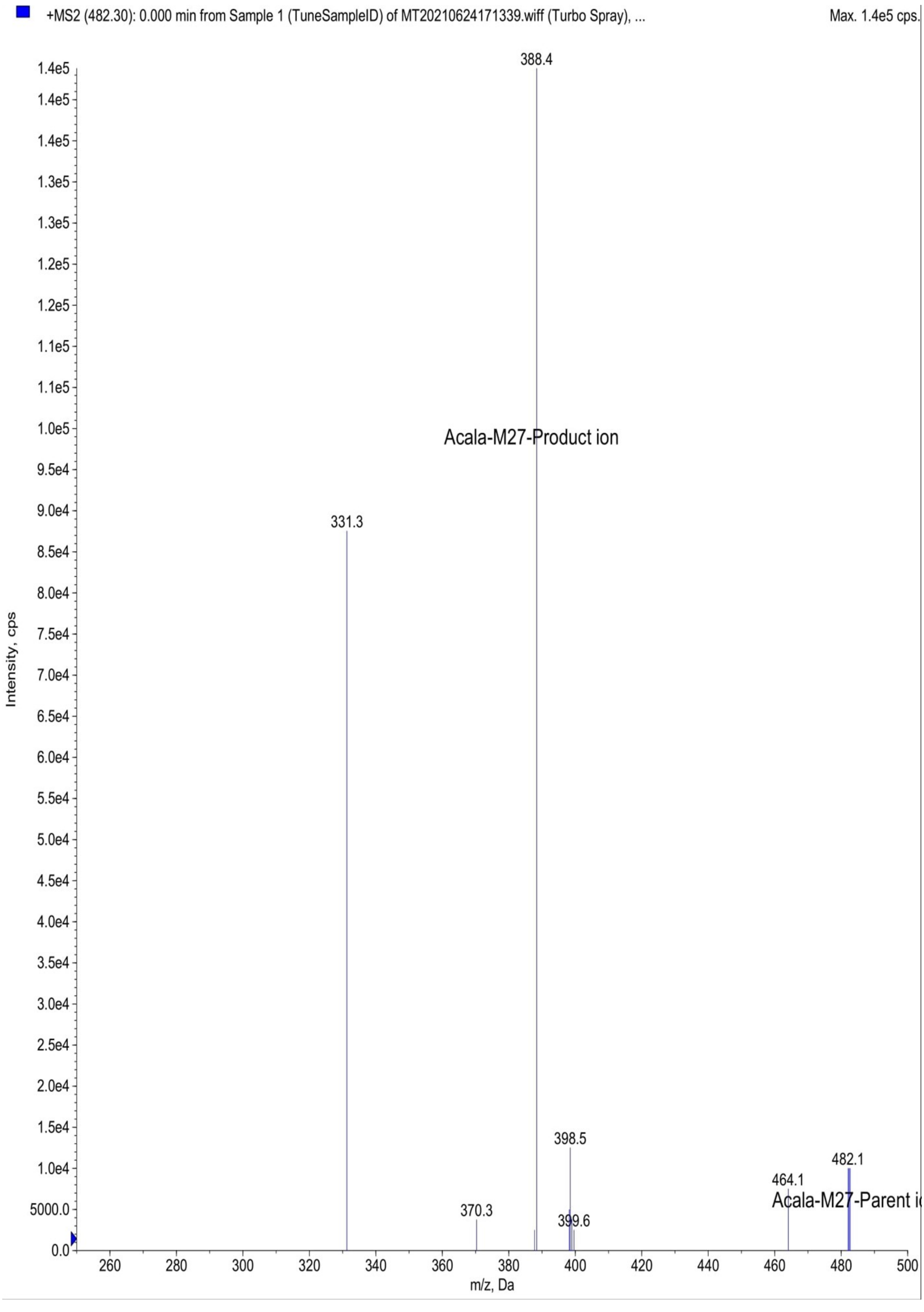

+MS2 (482.30): 0.000 min from Sample 1 (TuneSampleID) of MT20210624171339.wiff (Turbo Spray), ...
Max. 1.4e5 cps.
388.4
Acala-M27-Product ion
331.3
398.5
370.3
399.6
464.1
482.1
Acala-M27-Parent i
Intensity, cps
m/z, Da

C)

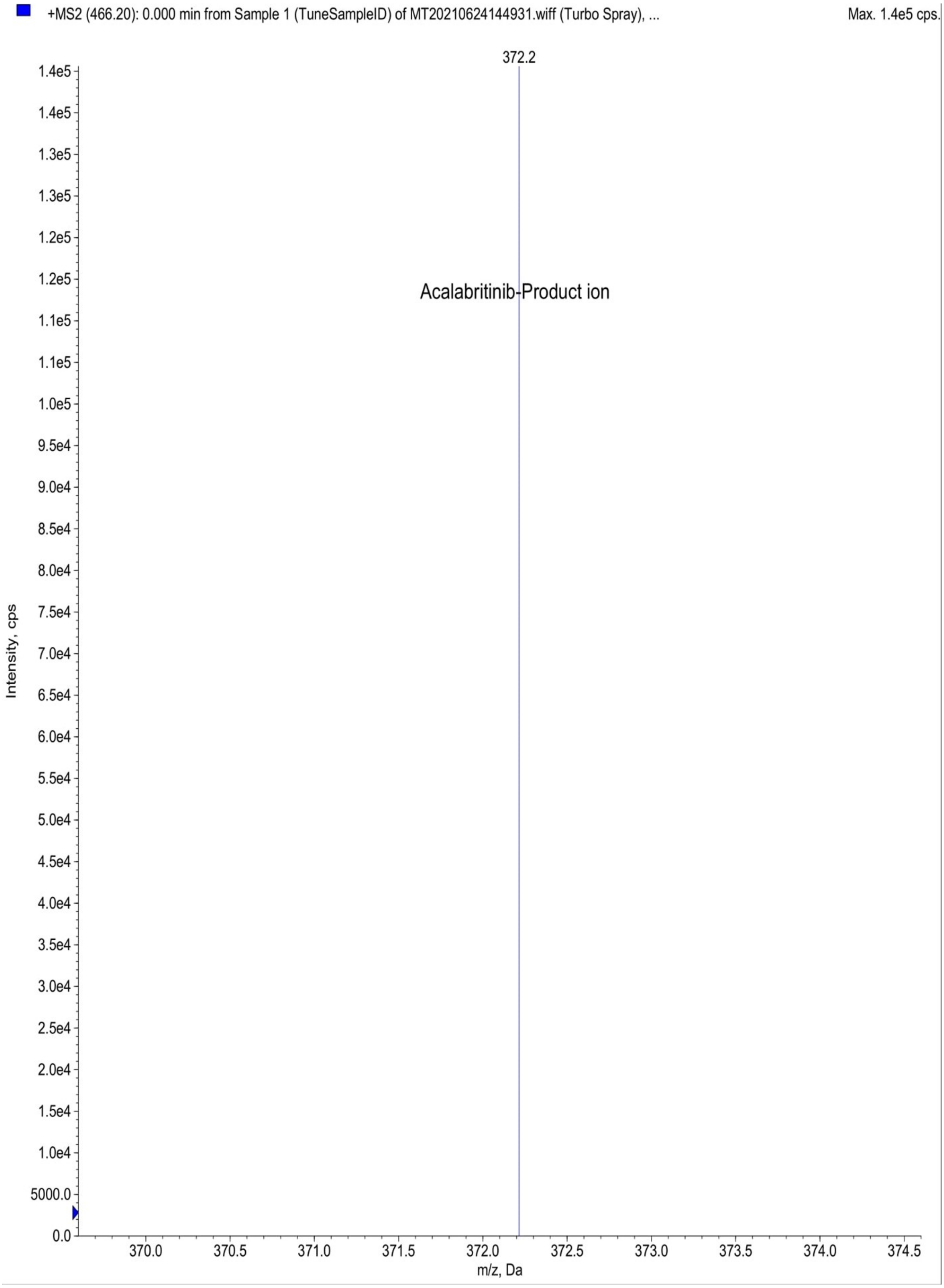

D)

Product ion mass spectrum for A) Acalabrutinib-D4, B) Acalabrutinib-M27-D4, C) Acalabrutinib-M27 and D) Acalabrutinib.
